# Yield, Quality, and Antioxidant Activity of Clove (*Syzygium aromaticum* L.) Bud Oil at the Different Phenological Stages in Young and Mature Trees

**DOI:** 10.1155/2020/9701701

**Published:** 2020-06-02

**Authors:** Faisal Nur Alfikri, Rini Pujiarti, Mochamat Gunawan Wibisono, Eko Bhakti Hardiyanto

**Affiliations:** Faculty of Forestry, Universitas Gadjah Mada, Jl. Agro, No. 1, Bulaksumur, Yogyakarta 55281, Indonesia

## Abstract

Buds and flowers of clove (*Syzygium aromaticum* L.) are economically important essential oil sources. The purpose of this study was to assess the yield, quality, and antioxidant activity of Zanzibar clove bud oil between three phenological stages (budding-3, full-budding, and flowering) in young (3-4 years) and mature trees (45 years). Chemical compositions of the oil were analyzed using GC-MS, and the physicochemical properties were measured based on SNI 06-4267-1996. Antioxidant activity was analyzed using the DPPH method. The results showed flowering buds of young trees produced higher yield (16.73%) than that of the mature ones (14.93%). The GC-MS analysis showed that the main bioactive compound of clove oil was eugenol (68.05–82.38%), which is highest at the flowering stage in mature trees. Almost all of the clove bud oils met physicochemical properties standard required by the SNI 06-4267-1996. DPPH scavenging activity IC_50_ ranged 15.80–108.85 *µ*g/mL, with the highest antioxidant activity at the flowering stage of young trees. The results indicate that clove at flowering stage produced the best essential oil ingredient, as well as the most efficient source of natural antioxidants with slight differences in quality between the young and mature trees.

## 1. Introduction

Clove (*Syzygium aromaticum* L.) buds and flowers are utilized for its essential oil. In Indonesia, clove is classified into four main varieties: Siputih, Sikotok, Zanzibar, and Ambon [1]. The Zanzibar variety produces more buds than other varieties and considered the most superior and cultivated variety [2]. A clove bud is a single flower with a length of 1-2 cm, while a clove flower has eight stages of clove bud development, namely young bud, budding-1, budding-2, budding-3, full-budding, flowering, initial fruiting, and full fruiting [2, 3].

Apart from the utilization of clove flowers in the cigarette industry, clove also produces essential oil with good yield ranging from 10% to 20%. Generally, the buds contain higher oil yield than the branches (5–10%) or leaves (1–4%) [1]. The phenological stage influences the yield and quality of clove bud oil. Razafimamonjison et al. [3] found that the full-budding stage, from buds collected from Madagascar, had the highest oil yield, while the full-fruiting stage had the lowest; and the eugenol content tended to increase from young bud stage to full-fruiting stage, i.e., from 72% to 82% [3].

Eugenol is the main component of clove oil, which has strong antioxidant activity [4]. Antioxidant is an important property, which is able to reduce impact of free radical activity [5]. Recently, additives that have antioxidant activity, such as butylated hydroxyl anisole (BHA) and butylated hydroxytoluene (BHT), are mostly used as an ingredient of medicine. However, these synthetic additives are potentially carcinogenic. Therefore, natural antioxidants such as that contained in clove oil are more desirable as they are safe and easy to obtain [4].

Young trees at 1–5 years old had unstable growth stage, while mature trees at 30–50 years old had stable growth stage [2,6]. There are obvious physiological differences between unstable and stable growth stage. However, studies on its yield and quality as essential oil are limited. Particularly, young trees at 3-4 years old had the first flowering period [7], which makes the flower buds an interesting material for experiment.

The flower bud at full-budding stage has the highest economic value compared with those at pre- and post-full-budding stage, which are also considered of lower quality. Harvesting of flower buds at early and late flower stage is avoided due to suspected low quality of flower. For this reason, most studies on clover oil have focused on full-budding stage. Lack of information on the physicochemical properties and biochemical activities of clove bud oil has minimized the use of pre- and post-full-budding buds as a source of bud oil production. Therefore, this study was conducted with the objective of assessing the yield, physicochemical properties, chemical composition, and antioxidant activity of clove oil extracted from flower buds taken at several budding stages of young (3-4 years old) and mature (45 years old) trees.

## 2. Materials and Methods

### 2.1. Flower Bud Collection and Distillation

The Zanzibar variety clove (*S. aromaticum* L.) buds were collected from the clove plantation owned by PT Cengkeh Zanzibar located in the village of Kalisidi, West Ungaran District, Central Java, Indonesia (573 m above sea level). Flower buds for each of three different phenological stages (budding-3, full-budding, and flowering) (Table 1) were harvested from 9 plants at 3-4 years old and 3 trees at 45 years old. Three replications were used for each phenological stage in young (3-4 years old) and mature (45 years old) trees, which every replication contains 200 ± 0.05 gr dry weight of clove buds.

The flower buds were grouped by phenological stage in each tray before placed under sunlight directly and dried for ±8 days or until 12%–14% of water content present [1]. Samples collected at each phenological stage in young and mature trees were hydrodistilled in 1500 mL water using the Clevenger apparatus for 8 hours. There was an addition of 500 mL water for every 2 hours during the extraction. The distilled bud oils were stored at 0°C until further analysis. The oil yield was calculated using the following formula:

(1) $$\begin{array}{c} \text{oil yield}\left(\%\right)=\frac{\text{specific gravity of oil x volume of oil}}{\text{dry air weight of bud}}\times \;100. \end{array}$$

### 2.2. Gas Chromatography-Mass Spectrometry (GC-MS) Analysis

Samples were diluted with analytical grade n-hexane with 1 : 1000 (oil: n-hexane) ratio prior to injection. The chemical components of clove oil were analyzed using GC-MS QP2010S at 70 eV and 310°C with HP Agilent column 5 (30 m × 0.25 mm with a film thickness of 1.0 *µ*m). Oven temperature was from 70°C for 5 minutes and programmed heating from 70°C to 300°C for 19 minutes, and injector temperature 270°C. Helium gas was used as a carrier at a constant flow rate of 0.50 mL/min, split ratio 80, and ion source temperature 225°C. The distilled oil from all bud stages was analyzed using GC-MS with a retention time of 60 minutes [8]. Chromatograms were analyzed based on the suitability of mass spectrometry and the NIST11 Library.

### 2.3. Physicochemical Properties Analysis

The physicochemical properties were analyzed based on SNI 06-4267-1996 [9], which have several parameters such as specific gravity, optical rotation, refractive index, and miscibility in 70% ethanol.

Specific gravity was measured using a pycnometer based on the ratio of the weight of oil and distilled water at the same temperature and volume. First, the weight (Mb) of the empty pycnometer was measured. Then, 5 mL distilled water was poured into the empty pycnometer, and its weight (M1) was measured. The distilled water was then removed, and the pycnometer was dried. The weight of the dried pycnometer was measured once more (Ma). Then, 5 mL of oil was put into the dried pycnometer and measured its weight (M2). The specific gravity at 15°C was calculated using the following formula:

(2) $$\begin{array}{c} \text{specific gravity}\left(15^{{}^{\circ}}\text{C}\right)={\text{BJ}}^{\prime }+0.00085\;\left(t1-t\right), \end{array}$$

where BJ′ is the specific gravity at room temperature, while *t*1 is the oil temperature at the time of measurement, and *t* is the standard oil temperature based on SNI (15°C). The value of 0.00085 is a correction factor. The specific gravity in the room temperature was calculated using the following formula:

(3) $$\begin{array}{c} \text{specific gravity}=\frac{\left(\text{M}2-\text{Ma}\right)}{\left(\text{M}1-\text{Mb}\right)}, \end{array}$$

where M1 is the weight of the pycnometer and distilled water (g), M2 is the weight of the pycnometer and oil (g), Ma is the empty pycnometer without oil (g), and Mb is the empty pycnometer without distilled water (g).

Optical rotation was measured using a polarimeter (WGX-4; Shanghai Benson Instrument Co. Ltd, Shanghai, China). Distilled water was added to the tube, and the area of polarization assessed. Oil was then added after removing the distilled water. The polarization field rotated to obtain a clear field of observation. The polarization angle was the difference between the slant of oil polarization and distilled water and calculated by the following formula:

(4) $$\begin{array}{c} \text{oil optical rotation }=\text{ pseudo oil optical rotation }-\text{ distilled water optical rotation}. \end{array}$$

Refractive index was measured using a refractometer (2WAj series, ABBE brand). The oil was dropped on the prism plane, and the cover was closed. The refractometer scale knob was rotated until the dark and bright limits hit the cross line intersection. The scale was determined from the index pointed by the scale line.

Miscibility in 70% ethanol was measured based on the volume ratio of oil in it. Moreover, 1 mL of oil was added with 1 mL of 70% ethanol, and the solution was shaken. The addition of ethanol continued until the oil completely dissolved (the amount of 70% ethanol addition was noted). Miscibility in 70% ethanol was calculated using the following formula:

(5) $$\begin{array}{c} \text{miscibility in }70\%\text{ ethanol }=\;\left(1\text{ ml oil}\right):\left(\text{ml }70\%\text{ ethanol}\right). \end{array}$$

### 2.4. Antioxidant Activity Analysis

The antioxidant activities were analyzed using the DPPH method based on Gülçin et al. [10] with a slight modification. Additionally, 3.5 mL of DPPH (Sigma-Aldrich, USA) of 0.1 mM was mixed with 0.5 mL of oil, which diluted with analytical grade ethanol at various concentrations (15–45 *µ*g/mL). The oil-DPPH solution was shaken and stored in a dark room for 30 minutes at 22°C. Blanks without additional oil in DPPH solution were prepared, and the UV-Vis spectrophotometer at a wavelength of 517 nm was used to measure the absorbance. The percentage of inhibition was calculated using the following formula:

(6) $$\begin{array}{c} \text{inhibition}\left(\%\right)=\frac{\left(A_{0}-A_{1}\right)}{A_{0}}\times \;100\%, \end{array}$$

where *A*_0_ and *A*_1_ are the absorbances of blank and DPPH solution with oil addition, respectively. The results were turned into a curve, and the antioxidant activity was presented with an IC_50_ value (inhibitory concentration to reduce DPPH radical to 50%). All treatments were replicated three times, and the data were presented in an average value.

### 2.5. Statistical Analysis

The collected data were analyzed using ANOVA and descriptive analysis. Factors with significant differences were further tested with the honestly significant difference (Tukey's HSD) at 5% test level using the SPSS software.

## 3. Results and Discussion

### 3.1. Oil Yield

**Figure 1:**
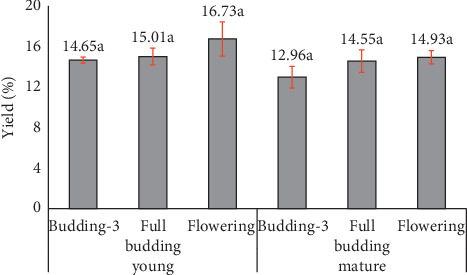
Yield of clove oil from three replications. Same letter notation (a, b, etc) indicates no significant difference (*p* > 0.05) in each age group.

The buds taken from young trees had higher oil yield than mature trees, and the oil yield increased with the maturity of stages, with flowering stage showing the highest oil yield (Figure 1).

The highest oil yield in young trees was also reported in several previous studies on other plant species [11, 12]. Composition of the vacuole structures led to high oil yield in young clove trees. Essential oil is one of the secondary metabolites stored in plant vacuoles [13]. Small vacuoles merge to form a single large structure as the plants grow [14]. The growth of cell size makes the vacuoles absorb more water to maintain cell turgidity during the life span of the plant [15]. In general, young trees have small vacuoles arranged densely, while mature ones have one large vacuole with higher water content. The high amount of water content in mature tree vacuoles makes it possible to decrease the yield of clove oil.

The bud at flowering stage was morphologically different from the budding-3 and the full-budding stages where at flowering stage the bud no longer has petals and stamens. The absence of petals and stamens was found to reduce the oil content by 5%–10% [7]. In contrast, the present study found that the oil yield at the flowering stage was higher than other stages due to the maturity of the oil cells.

The bud at flowering stage has idioblast composition with bigger bubble stadium than that at full-budding stage. In the meantime, the bud at full-budding stage has idioblast composition with bubble stadium bigger than that at budding-3. The higher oil yield of bud at flowering stage than that at budding-3 stage was likely due to the biosynthesis characterization by the existence of secretory tissues such as trichomes, osmofore glands, and oil cells or idioblasts [14]. The oil cells have three development stages, including oil droplets stage, membranogenous droplets with the cupula, and oil drops with the cupula. This cell development continues until the droplets grow larger and turn into bubbles and fill the cell lumen to form idioblasts [16]. The biosynthesis starts from the first budding stage, meaning the transformation of the droplet into bubble occurs along with the budding stages [17].

### 3.2. Chemical Composition

The GC-MS analysis was able to identify 11-12 compounds in clove oil from young trees (age 3-4 years) and 9–12 compounds in clove oil from mature trees (age 45 years) in each phenological stage (Table 2). The oil at flowering stage has the most complex chemical composition. The identified main compound of clove oil included eugenol, eugenol acetate, and *β*-cis-caryophyllene. The bud at flowering stage of young and mature trees contained 81.3% and 82.3% eugenol, respectively, while that at budding-3 stage of the young and mature trees contained 10.3% and 15.5% eugenol acetate, respectively. The highest *β*-cis-caryophyllene content (7.70%) was obtained from the bud at budding-3 stage of the young trees, while the mature ones had 5.57% of *β*-cis-caryophyllene content.

The percentage of eugenol at all three budding stages in mature trees was higher than that in the young ones, mainly due to differences in age; mature trees (45 years old) are in stable growth stage (have passed the critical stage of growth) and therefore produce more flowers. In a good budding period, all assimilates and mineral nutrients are translocated to flowers to facilitate their growth and development [18]. The amount of assimilates and nutrients absorbed also affects the percentage of chemical composition.

Eugenol and eugenol acetate determine the quality of clove oil, while the hydrocarbon defines the aroma and typical properties of essential oil. The combination of *β*-cis-caryophyllene and eugenol results in a bitter taste and spicy aroma [6,19]. *β*-cis-caryophyllene is a hydrocarbon of sesquiterpene fraction [7], which produces a stronger aroma. The stronger aroma is often demanded in the cigarette industry, so that the flowering stage is considered of lower value due to its low *β*-cis-caryophyllene content. Conversely, the bud at flowering stage produces essential oil with high eugenol and more complex chemical composition.

### 3.3. Physicochemical Properties

Phenological stages had a significant effect on the refractive index (*p* < 0.05). The bud at flowering stage had the highest refractive index of 1.54. In general, the specific gravity of clove oil produced by young trees (1.06–1.07) was not different from mature trees (1.05–1.07). There were no considerable differences between the value of miscibility in ethanol in young and mature trees. The value of miscibility in ethanol at flowering stage in young trees was higher than that in the mature ones.

The oil from young and mature trees met the Indonesian National Standard (SNI 06-4267-1996) [9] for specific gravity, refractive index, and miscibility in 70% ethanol in all phenological stages, whereas that of full-budding and flowering stages in mature trees met the optical rotation standard (Table 3).

Specific gravity of the compound constituent, such as eugenol (1.0651), eugenol acetate (1.0806), and *β*-cis-caryophyllene (0.9075) [6, 20, 21] affected the specific gravity of the oil. Young trees tended to yield oil with lower specific gravity at all bud stages due to the changes of the long carbon chains and high molecular mass compounds such as *β*-cis-caryophyllene and eugenol acetate, compared with mature trees; a similar finding was also reported in a previous study [3]. The raise in the compound complexity of the oil at flowering stage seems to be the cause of specific gravity increase.

Optical rotation of oil extracted from young trees was higher than that from mature trees, which was similar to that found in eucalyptus oil [12]. According to Pujiarti et al. [11], tree age has a diverse influence on the optical rotation of essential oils. Changes in the polarization angle increase with the decrease in the viscosity of the material [22]. The results of the chemical composition analysis showed several long carbon chains and high molecular masses compounds such as isolongifolene, *β*-cis-caryophyllene, *α*-humulene, eugenol acetate, and caryophyllene oxide contain clove oil. The compounds affect the viscosity and the rotational angle of polarization.

Refractive index average of the oil from young trees was lower than that from the older ones, especially for the flower bud at the budding-3 and full-budding stages. Similar results were also reported in previous studies on eucalyptus oil, indicating that a 10-year-old tree had a lower refractive index than a 15-year-old tree [11]. The low eugenol content in young trees influences the refractive index, namely 1.5405 at 20°C [21]. This figure exceeded the SNI 06-4267-1996 standard [9]. A mixture of other chemical compounds and water might also be the cause of the decline in the refractive index. The more the water content, the smaller the refractive index value [23]. The refractive index value also affects the colour of the oil. For instance, a clear oil has a higher refractive index [11]. Each budding stage yielded different oil colour; flowering-stage buds produced clearer oil compared with other stages.

The value of miscibility in ethanol from the bud at flowering stage of young trees was higher than that of other stages (full-budding stage had the lowest value). The high hydrocarbon content such as psi-cumene; hemimellitene; p-cymene; 3-ethyl-o-xylene; 4-ethyl-o-xylene; 1,2,3,4-tetramethylfulvene; and prehnitene influenced the miscibility in 70% ethanol. The content of terpenes and oxygenated hydrocarbons influenced miscibility in ethanol. The higher content of terpenes makes oil less soluble, and the higher content of oxygenated hydrocarbons makes oil more soluble [6]. Clove oil from all stages and taken from trees of different ages met the SNI 06-4267-1996 standard [9]. The flower at budding-3 stage taken from mature trees had the highest miscibility in ethanol.

### 3.4. Antioxidant Activity

**Figure 2:**
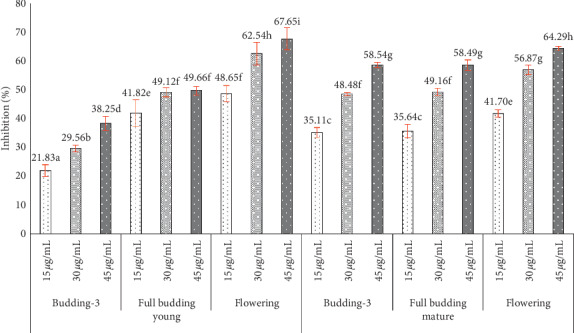
DPPH radical inhibition (%) of clove oil at different ages, phenological stages, and concentrations from three replications (*p* < 0.05).

Percentages of DPPH radical inhibition by oil at three concentrations are shown in Figure 2. Interactions between tree age, phenological stage, and oil concentration for antioxidant activity were significant (*p* < 0.05). The flowering stage extract of young trees at a concentration of 45 *μ*g/mL (67.65%) had the highest antioxidant activity. In mature trees, the extract of bud at flowering stage at a concentration of 45 *μ*g/mL (64.29%) had the highest antioxidant activity. In general, young trees have antioxidant activity greater than mature ones. The antioxidant activity increased with the budding stage.

**Figure 3:**
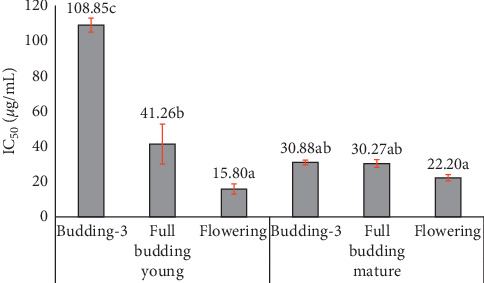
DPPH IC_50_ (inhibitory concentration to reduce DPPH radical by 50%) of clove oil at different ages and phenological stages from three replications (*p* < 0.05).

Phenological stages had significant effect on the ability to inhibit 50% of free radical activity. The extracts of bud at full-budding and flowering stages from mature trees had nearly the same IC_50_ value (Figure 3). Moreover, the extract of bud taken at flowering stage from mature and young trees had the highest ability to inhibit 50% of DPPH radical activity.

The bud at flowering stage produced oil with the highest antioxidant level probably due to the high eugenol content. The assertion was in line with the results of the chemical composition analysis, which showed flowering-stage buds produced more eugenol than the budding-3 and full-budding stages. According to Razafimamonjison et al. [3], flowering-stage buds had higher eugenol content than the others and therefore had higher antioxidant levels than other stages. Other studies showed clove oil has antimicrobial and antioxidant activity due to the presence of eugenol and other phenolic compounds [24]. Previous research also showed eugenol had a higher antioxidant activity than synthetic substances such as butylated hydroxyanisole (BHA) [25]. Eugenol is one of the phenolic compounds with an aromatic ring. This structure allows phenolics to stabilize free radicals by transferring hydrogen atoms to radicals, as it is able to stabilize itself due to its resonant structure [26].

Young trees produced oil with the highest antioxidant level due to the high phenolic content. According to the result of the chemical composition analysis, buds at flowering stage from young trees contained a higher percentage of phenolic compounds such as 2-indanol, eugenol, and eugenol acetate than those from mature trees, which was possibly to influence the value of antioxidant levels.

## 4. Conclusions

Clove flower buds at flowering stage had the highest yield, eugenol content, and refractive index. The main components of clove essential oils were eugenol, *β*-cis-caryophyllene, and eugenol acetate. The extract of buds at flowering stage in mature trees met all criteria in the Indonesian National Standard (SNI). Best quality of clove essential oil was obtained from buds of mature trees at flowering stage, while those of young trees had the strongest antioxidant activity. The bud of the Zanzibar clove variety taken at flowering stage produced the best essential oil ingredient and a source of natural antioxidants, with some differences in quality between young and mature trees. This finding was of importance in the selection of clove buds from different budding stages. Further studies are suggested to focus on the use of clove buds in two critical stages, namely the development of a better distillation method to improve oil quality and the application of clove essential oil as a natural antioxidant.

## Figures and Tables

**Table 1:** Clove bud on three phenological stages.

Phenological stage	Photo	Colour	Wet bud length (mm)	Other traits
Budding-3 (4^th^ stage)	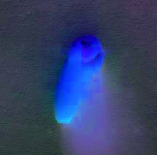	Yellowish green	13.3	Small cap and half-covered by its calyxes
Full budding (5^th^ stage)	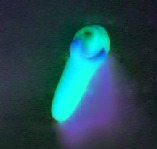	Yellow to orange	20.8	Round cap and full, uncovered by its calyxes
Flowering (6^th^ stage)	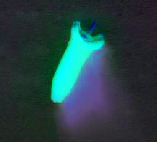	Red	19.4	The cap is lost (bloomed) with only calyxes and stamens left

Bud stage determination follows Razafimamonjison et al. [3].

**Table 2:** Chemical composition and concentration of clove bud oil at different bud stages and tree ages.

No.	RT^∗^	SI^∗∗^	Compound name^∗∗∗^	Chemical formula	Concentration (%)					
					Young trees			Mature trees		
					Budding-3	Full budding	Flowering	Budding-3	Full budding	Flowering
1.	5.166	76	Mesitylene	C_9_H_12_	—	—	—	—	0.93	—
2.	5.916	95	Psi-cumene	C_9_H_12_	3.72	3.58	2.93	2.01	3.03	2.50
3.	6.810	84	Hemimellitene	C_9_H_12_	1.58	1.69	1.24	0.81	1.29	1.14
4.	7.658	70	2-Indanol	C_9_H_10_O	—	—	1.15	—	—	0.32
5.	7.885	80	p-Cymene	C_10_H_14_	0.53	0.70	1.35	—	1.18	—
6.	8.484	86	3-Ethyl-o-xylene	C_10_H_14_	0.98	1.05	-	0.67	—	1.42
7.	8.709	91	4-Ethyl-o-xylene	C_10_H_14_	2.25	2.30	1.75	1.10	2.14	1.51
8.	9.631	87	1,2,3,4-Tetramethylfulvene	C_10_H_14_	1.76	2.10	1.42	1.04	2.02	1.22
9.	9.762	91	Prehnitene	C_10_H_14_	2.17	2.87	1.67	1.44	2.73	1.68
10.	10.329	49	Isolongifolene	C_15_H_24_	0.99	—	—	—	—	—
11.	16.222	96	Eugenol	C_10_H_12_O_2_	68.05	74.62	81.33	71.89	77.61	82.38
12.	17.653	90	*β*-cis-Caryophyllene	C_15_H_24_	7.70	4.46	2.44	5.57	2.53	3.21
13.	18.447	60	*α*-Humulene	C_15_H_24_	—	—	0.21	—	—	0.39
14.	20.013	89	Eugenol acetate	C_12_H_14_O_3_	10.26	6.62	4.32	15.47	6.54	3.93
15.	21.290	61	Caryophyllene oxide	C_15_H_24_O	—	—	0.18	—	—	0.30

Total					100.00	100.00	100.00	100.00	100.00	100.00

^∗^RT: retention time (minute); ^∗∗^SI: similarity index based on mass spectrometry. Compound names were identified ^∗∗∗^by NIST11 Library. The results were average from three replications.

**Table 3:** Physicochemical properties of clove bud oil in different ages and phenological stages.

Test	SNI 06-4267-1996	Age	Phenological stage	Value
Specific gravity 15°	1.04 to 1.07	Young	Budding-3	1.07 ± 0.01
			Full budding	1.07 ± 0.00
			Flowering	1.06 ± 0.00
		Mature	Budding-3	1.05 ± 0.01
			Full budding	1.06 ± 0.01
			Flowering	1.07 ± 0.00
Optical rotation (*α* D)	−1°35' to 0°	Young	Budding-3	0°17' ± 0°85'
			Full budding	0°60' ± 0°45'
			Flowering	0°83' ± 0°80'
		Mature	Budding-3	1°63' ± 1°10'
			Full budding	−0°40' ± 1°40'
			Flowering	−0°23' ± 1°08'
Refraction index (nD) 20°	1.529 to 1.537	Young	Budding-3	1.534 ± 0.002
			Full budding	1.534 ± 0.001
			Flowering	1.537 ± 0.000
		Mature	Budding-3	1.535 ± 0.001
			Full budding	1.535 ± 0.001
			Flowering	1.536 ± 0.001
Eugenol (%)	80 to 95	Young	Budding-3	68.05
			Full budding	74.62
			Flowering	81.33
		Mature	Budding-3	71.89
			Full budding	77.62
			Flowering	82.38
Miscibility in 70% ethanol	1 : 2 clear, clear afterwards	Young	Budding-3	1 : 1–1 : 2
			Full budding	1 : 2
			Flowering	1 : 1–1 : 2
		Mature	Budding-3	1 : 1–1 : 2
			Full budding	1 : 2
			Flowering	1 : 2

SNI: *Standard Nasional Indonesia* (Indonesian National Standard) [9]. The results were average from three replications.

## Data Availability

All data generated or analyzed during this study are available on request through the corresponding author at rpujiarti@ugm.ac.id.
